# Climate Change, Epigenetics, Microbiota, and Health

**DOI:** 10.3390/ijerph23030388

**Published:** 2026-03-18

**Authors:** Francesco Misiti, Alessandra Sannella

**Affiliations:** 1 Human, Social and Health Department, University of Cassino and Lazio Meridionale, V. S. Angelo, Loc. Folcara, 03043 Cassino, Italy; alessandra.sannella@unicas.it; 2 European University of Technology, EUt+, European Union

**Keywords:** climate change, microbiota, epigenetics, non-communicable diseases (NCDs), cardiovascular diseases, respiratory diseases, public health, adaptation, one health

## Abstract

**Highlights:**

**Public health relevance—How does this work relate to a public health issue?**

- Climate change acceleration exacerbates non-communicable diseases (NCDs) like cardiovascular and respiratory diseases.
- Vulnerable populations face heightened risks from environmental pressure.

**Public health significance—Why is this work of significance to public health?**

- This work clarifies the molecular pathways linking climate stressors to NCDs.
- Multi-omics technologies are essential for understanding the complex relationships between climate stressors and NCDs.

**Public health implications—What are the key implications or messages for practitioners, policy makers, and/or researchers in public health?**

- Epigenetic and microbiota biomarkers are needed to plan adaptation strategies against climate change-driven NCDs.
- Transdisciplinary public health strategies underscore the critical importance of the One Health framework in reducing health inequalities.

**Abstract:**

The acceleration of climate change poses a growing threat to human health, particularly by exacerbating non-communicable diseases (NCDs) such as cardiovascular and respiratory conditions. Rising global temperatures amplify air pollution and environmental toxins, disproportionately affecting vulnerable populations. This narrative review explores the complex pathways linking climate-related environmental stressors to adverse health outcomes, focusing on the intermediary roles of epigenetic modifications and alterations in the microbiota. Epigenetic processes, including DNA methylation and histone modifications, may mediate how environmental exposures influence gene expression and disease susceptibility. Concurrently, changes in microbiota composition induced by pollutants and temperature fluctuations can promote inflammatory responses and immune dysfunction. Elucidating these molecular mechanisms is essential for developing targeted interventions and adaptive strategies to mitigate the health impacts of climate change. This review underscores the importance of identifying epigenetic and microbiota-based biomarkers for early risk stratification and for informing public health prevention and adaptation policies. A transdisciplinary approach, grounded in the One Health framework, is critical to addressing the growing burden of climate-sensitive diseases and reducing health inequalities.

## 1. Introduction

Climate change, marked by extreme events such as heatwaves, droughts, wildfires, and glacier melting, puts environmental pressure on global population health [1]. The Intergovernmental Panel on Climate Change (IPCC) has established that these phenomena exacerbate morbidity and mortality, particularly through rising temperatures, air pollution, and exposure to pollutants, with direct impacts on vulnerable people [2,3]. Non-communicable diseases (NCDs), chronic conditions including cardiovascular, respiratory, neurodegenerative diseases, and skin cancers [4], are the leading global cause of death and disability, responsible for over 40 million annual deaths (71% of the total deaths worldwide). Emerging evidence indicates that climate-related environmental stressors adversely affect both cardiovascular and respiratory health [1]. These findings suggest a complex interplay between climate change and cardiovascular and respiratory diseases, mediated by diverse biological mechanisms that remain poorly understood. In this context, epigenetic alterations and microbiota dysbiosis emerge as key mediators; environmental stressors induce epigenetic changes that alter gene expression without modifying the DNA sequence, heightening NCD susceptibility, while temperature fluctuations and pollutants disrupt microbial composition, promoting inflammatory responses and intestinal permeability [5,6]. These mechanisms interconnect, as microbial short-chain fatty acids (SCFAs) influence epigenetic processes, amplifying climate effects on cardiovascular and respiratory health [7]. Various factors influence NCDs and share similar molecular changes across different organs. This review reports on the role of epigenetics alterations and the impact of host–microbiota interaction in the context of climate change, and although almost all of the alterations reported in the literature refer to a large panel of climate effects, we focus on suboptimal temperatures and air pollution; clarifying these molecular mechanisms and their interactions, along with the development of new analysis methodologies to study them, could help us understand the adverse effects of climate change on human health and plan future mitigation strategies. We have outlined terms that are frequently used in this review and in the literature in Tables S1 and S2.

## 2. Materials and Methods

A literature search was conducted via the PubMed and Scopus databases to identify studies assessing how climate change can drive disease through its impact on the gut microbiota and epigenetic modifications (Table 1). We focused on the most well-studied environmental factors associated with climate change, including air pollution and heath waves and the following keywords were used in various combinations: “climate change”, “health” OR “healthy”, “microbiota” OR “gut microbiota”, “epigenetics” OR “epigenetic”, “cardiovascular diseases”, “respiratory diseases” OR “non-communicable diseases” from the beginning of 2019 to the end of 2025. We included English language-based, international peer-reviewed articles and electronic books. We employed a snowballing search methodology, utilizing the references cited in the articles identified through the literature search. Each identified item was assessed for relevance by the authors.

## 3. Climate Change and Health

Climate change drives health risks primarily through two interconnected mediators: rising global temperatures (including heatwaves and other extreme events) and worsened air pollution, which, in turn, indirectly trigger epigenetic modifications and microbiota dysbiosis underlying non-communicable diseases (NCDs). Recent research has linked epigenetics to cardiovascular (CVDs) and respiratory diseases [8,9]. These pathways are supported by epidemiological evidence showing increased cardiovascular and respiratory mortality [10]. Anthropogenic air pollution, which is both a driver and a consequence of climate change, intensifies with warming through enhanced atmospheric reactivity and increased wildfire activity, elevating levels of fine particulate matter (PM_2.5_, PM_10_) [1]. PM_2.5_, O_3_, and NO_2_ exposure is responsible for 6.7 million annual deaths, with short-term spikes raising stroke and myocardial infarction risk by 5–10% [11,12,13,14,15]. These pollutants are closely linked to climate change through the combustion of fossil fuels [1], triggering systemic inflammation, oxidative stress, and endothelial dysfunction, accelerating DNA methylation clocks, and decreasing gut beneficial bacteria, leading to a rise in pro-inflammatory taxa with serious health consequences. Pollutants, including particulate matter (PM) and chemical gases such as nitrogen oxides (NOx), translocate from the lungs to the bloodstream and the gut, disrupting microbiota via oxidative damage and reducing short-chain fatty acid levels, which, in turn, indirectly influences host epigenetics [16,17]. Bushfire smoke, particularly PM_2.5_ particles, has driven sharp increases in respiratory issues like asthma and COPD, as well as CVD effects in New South Wales and the Australian Capital Territory from 2019 to 2020, leading to more emergency department visits and hospitalizations for respiratory conditions, plus around 400 excess deaths overall [18]. Thunderstorm asthma events further highlight acute respiratory dangers. In Melbourne, Australia, in November 2016, they caused 3365 excess emergency respiratory visits, about 2300 ambulance calls, and 9 deaths [19]. Similarly, in Yulin, China, in September 2022, peaks hit 1432 emergency asthma visits on the first day, with many subsequent hospitalizations. The majority (71.0%) were aged 40 years or younger, with half being children and adolescents (≤18 years), indicating vulnerability in younger populations [20]. Heatwaves significantly elevate cardiovascular disease (CVD) mortality risks, as shown in global meta-analyses from various locations between 1980 and 2021, with an overall +11.7% increased risk; this rises to +14.7% in people aged 65 and older, and is even higher in tropical climates [21]. In Europe across multiple sites during recent summers, each +1 °C rise during heatwaves correlates with +3.4% CVD mortality, alongside +1.6% for arrhythmias and +2.1% for cardiac arrest [22]. Global temperature rises exert direct effects despite core body temperature homeostasis, as skin and gut mucosal temperatures fluctuate with ambient heat, activating heat-shock responses, neuroendocrine stress, and intestinal barrier leakage, directly perturbing microbiota and epigenetics [23]. Body core constancy is maintained in the short term but fails under prolonged stress, amplifying NCDs [24].

### Public Health Implications

Among the secondary effects of accelerated climate change, in addition to the evident increase in cases of cardiovascular and respiratory diseases, there is an increase in the burden on health services in human, social, and economic terms, particularly during heat waves and pollution peaks [3]. The possibility of cross-referencing climate data with health data in early warning systems can serve both as a warning system and as a prevention and adaptation system. This can translate into a system of public health communication, hospital organizational resource planning, and adaptation and mitigation plans for workplaces and environments. Promoting recognition of these factors through indicators is a valuable tool, and is also in line with efforts to combat health inequalities, develop equitable adaptation strategies, and pursue social justice through the One Health approach. Furthermore, in line with the concept of pathoclima—diseases amplified by accelerating climate change and its cascading socio-environmental impacts, as defined by Buizza et al. [25]—climate-related exposures exacerbate syndemic burdens on vulnerable populations and the socio-health structure. The interpretative framework aligns with the One Health approach to the biological and social exposome, where social determinants of health influence health outcomes and underscore the urgency of equitable adaptation to counter growing inequality.

## 4. Pathological Mechanisms Related to Climate Change

### 4.1. Epigenetics

Environmental factors such as pollutants, diet, and lifestyle can influence the function, but not the structure, of certain DNA traits in individuals. Any factor that can modify the function of a gene can be considered an epigenetic factor. It has been shown that many diseases, including CVD and metabolic diseases, have an epigenetic basis [26,27]. The molecular mechanisms underlying epigenetic changes include DNA methylation, modifications of specific amino acids in proteins called “histones,” and alterations in chromatin structure. Collectively, these mechanisms can regulate the activation or deactivation of specific genes [28]. Research has revealed that levels of DNA methylation (DNAm) at many cytosine–phosphate–guanine sites (CpGs) are reliable indicators of molecular ageing, showing more potential than other markers of biological ageing [29]. The concept of epigenetic “clocks” indicates that accelerated molecular ageing may be a mechanism through which stressful psychosocial and physiological conditions contribute to adverse health outcomes [30]. Epigenetics is a molecular process that can directly alter phenotypic variation, potentially making it heritable in future generations [31]. Therefore, individuals with the same genotype may exhibit different phenotypic plasticity depending on their environment. Cardenas et al. [32] conducted the first review of epigenetic mechanisms related to various climatic factors, including temperature, humidity, and precipitation, as well as climate change-associated exposures such as natural disasters and malnutrition, and how these factors may be connected to allergic diseases. The authors highlight that temperature is the most widely studied factor, with findings suggesting that epigenetic modifications and accelerated epigenetic aging act as mediators between climate change and the rising prevalence of allergic diseases. The possibility of integrating epigenetic markers into longitudinal studies in populations most at risk of climate change impacts could improve forecasting models and guide prevention strategies for “personalised” or community-based medicine. The analysis of exposome factors and their associated methylation patterns could help develop targeted public health strategies.

#### 4.1.1. Epigenetic Studies and Suboptimal Temperature

Several studies have linked environmental temperature to epigenetic modifications in humans [33], identifying 15 differentially methylated genes in response to temperature exposure [31]. However, previous studies have primarily focused on the mean temperature during different exposure windows [34]. This study identified 14 differentially methylated CpGs and 70 DMRs (differentially methylated regions). Notably, a CpG site linked to the KCNK4 gene showed increased DNA methylation in response to temperature changes. Additionally, it has been demonstrated that medium-term exposure to high temperatures accelerates multiple epigenetic ageing clocks [35,36]. Higher average annual temperatures were linked to increased epigenetic ageing of biomarkers, particularly among women, obese individuals, and those with CVDs. This acceleration is significant, as it can indicate risks for all-cause mortality and diseases like asthma and allergic sensitisation in children [37].

#### 4.1.2. Epigenetic Studies of Wildfires and Air Pollution

A recent epidemiological study found that accelerated DNA methylation age is associated with an increased cardiovascular risk for individuals exposed to elevated traffic-related air pollution [38]. Air pollution derived from wildfires may also affect DNA methylation. Studies [39] have reported 26 CpGs and 33 differentially methylated regions associated with wildfire-related particulate matter. Another study showed that children exposed to wildfires had increased methylation in the FOXP3 promoter, suggesting decreased gene expression related to immune tolerance to allergens [40]. Both studies focused on DNA methylation in blood cells, while similar changes have also been observed in the nasal epithelial cells of rhesus macaques exposed to wildfire smoke [41]. These DNA methylation alterations induced by wildfire smoke disrupt gene expression, compromising immune and nervous system function and posing risks of reduced lung function, altered immune responses, and chronic respiratory diseases. Hypermethylation of immune genes, such as FOXP3, and of Th1 and Th2 pathways, weakens defenses against infections and cancer [42]. Persistent changes in nasal and sperm methylomes promote chronic inflammation, asthma, and pulmonary fibrosis [43]. Long-term effects include impaired neuronal development and diminished lung function in early-exposed primates [41].

### 4.2. Host–Microbiota Interaction

The gut microbiota is a complex component of the gastrointestinal tract, comprising over 100 trillion microorganisms. It encompasses bacteria, archaea, and eukarya and has evolved in tandem with its host to develop a mutually beneficial relationship. Approximately 85% of the total bacteria, including *Lactobacilli* and *Bifidobacteria*, are organisms that coexist with the host, while others, such as *Clostridium* and *Fusobacterium*, have the potential to become pathogenic [44]. Most bacterial species are categorised into the phyla *Firmicutes* (Gram-positive) and *Bacteroidetes* (Gram-negative), with the remainder belonging to *Actinobacteria*, *Fusobacteria*, *Proteobacteria*, and *Verrucomicrobia* [45,46]. Changes in the types of microbes living inside our bodies, especially in the gut, could be influenced by climate change [47,48].

#### 4.2.1. Microbiota and Suboptimal Temperature

A previous study suggests that temperature fluctuations can affect the composition and function of the microbiome in both humans and other animals [49]. For instance, studies have shown that exposure to high temperatures can alter the gut microbiome composition, leading to increased populations of bacteria associated with inflammation and metabolic disorders [50]. Moreover, recent findings highlight the crucial role of temperature in shaping microbial communities across various locations within the human body, including the skin, gut, and mouth. These results suggest that fluctuations in temperature, whether sudden or prolonged, can disrupt the delicate balance of microbial ecosystems in the body, potentially affecting immune response, metabolism, and overall well-being [51]. As climate change leads to more frequent and intense heat waves, studies on the microbiota could help develop strategies to adapt to these changes in human populations. Gaining insight into how organisms respond to heat stress through their microbiomes may inform efforts to enhance community resilience in the face of similar environmental challenges [52].

These changes may exacerbate the effects of climate change on our health or enable us to adapt more effectively to the changing environment. Our gut microbes play crucial roles in digesting food, producing essential nutrients, and maintaining the health of our intestines. So, any changes in the types of microbes in our gut could affect our health [53]. The *Firmicutes* to *Bacteroidetes* (F/B) ratio is often examined as a marker of health, with higher *Firmicutes* levels associated with better physical health. However, studies on this ratio have shown conflicting results, potentially due to methodological differences, subject characteristics, and lifestyle factors that can influence microbiota composition [54].

Risely et al. [55] observed a notable shift in the human gut microbiota, characterised by increased levels of *Bacteroides* and *Fusobacterium*, and decreased levels of lactic acid bacteria. Climate change’s impact on microbiota and immune health is partly due to heat stress, which triggers neuroendocrine activation, oxidative stress, and increased intestinal permeability. These changes, along with microbiota dysbiosis, increase gut permeability and inflammation. Recent studies have investigated the potential relationship between the gut microbiota and heart disease [56,57].

#### 4.2.2. Microbiota and Air Pollution

In 2019, Li et al. collected airborne microbiota from different sites in the city of Xiamen, China, demonstrating that exposure to ultrafine particles increased levels of *Proteobacteria* and *Firmicutes*, depending on the urbanization level [58]. These findings support the thesis that environmental pollutants, including ultrafine particles, PM_2.5_, SO_2_, and O_3_, can significantly alter the composition of the human gut microbiota [59].

Recent studies [60] have also provided strong evidence for the role of microbial metabolites in influencing host epigenetics and their subsequent physiological effects. For example, it has been demonstrated that SCFAs produced by gut microbiota play a crucial role in modulating the differentiation and function of immune cells through epigenetic modifications, underscoring the intricate relationship between microbial metabolites and host immune responses [57]. Therefore, the impact of gut microbiota on the host’s physiological responses to climate warming is intricate and still widely debated. Further research is necessary to fully grasp the connection between physiology and gut microbiota responses to higher temperatures.

Most gut microbiota studies have concentrated on the impact of rising average environmental temperatures on various animals [49]. The generation of a Human Gut Cell Atlas (HGCA) will provide a unique and valuable reference map, enhancing research on intestinal health and disease, as well as on the effects of climate change [61].

#### 4.2.3. Methodologies for Studying Epigenetic and Microbiota Alterations in Response to Climate Change

Research into epigenetic modifications as responses to climate change has become essential, shedding light on the molecular processes by which environmental stressors influence gene regulation in human populations. Epigenetic alterations, such as DNA methylation, histone modifications, and chromatin accessibility, are vital links between external climatic influences and genetic functions, potentially affecting human health, disease vulnerability, and adaptive capabilities [26,27]. Scientists employ various techniques across molecular biology, high-throughput sequencing, bioinformatics, and epidemiology to explore these modifications, each providing valuable insights into how climate-related challenges influence the human epigenome [62]. Whole-genome bisulfite sequencing (WGBS) is a primary method for examining DNA methylation changes, enabling researchers to discern methylation patterns with single-base precision across the genome. Other methods, including reduced representation bisulfite sequencing (RRBS) and methylation arrays such as the Illumina Infinium HumanMethylation450 and MethylationEPIC BeadChips, focus on specific CpG sites crucial for gene regulation, thereby enabling large-scale investigations of epigenetic modifications associated with environmental exposures. In addition to analysing DNA methylation, researchers utilise chromatin immunoprecipitation followed by sequencing (ChIP-seq) to study histone modifications that modulate transcription activity.

Additionally, the combined analysis of Transposase-Accessible Chromatin using sequencing (ATAC-seq) and RNA sequencing (RNA-Seq) is considered a powerful approach for single-omic studies to systematically elucidate the mechanisms underlying climate-induced stress responses [63,64]. Additionally, quantitative polymerase chain reaction (qPCR) and digital droplet PCR (ddPCR) remain vital for validating high-throughput screening results, providing high sensitivity and specificity for confirming differentially methylated regions and changes in gene expression [63]. The synthesis of extensive epigenomic data with sophisticated bioinformatics tools is essential for analysing climate-induced epigenetic shifts. Statistical strategies, including differential methylation analysis, pathway enrichment analysis, and network-based modelling, help researchers pinpoint key epigenetic markers associated with climate stressors. Machine learning techniques and AI-based methods have revealed intricate patterns within complex multidimensional datasets related to epigenomics, metagenomics, and environmental factors, thereby improving the predictive modelling of health outcomes linked to climate change [65]. Such methodologies help evaluate potential transgenerational epigenetic effects, as maternal exposure to climate-related stressors has been shown to influence offspring DNA methylation profiles [66]. Other studies have quantified CpG-site methylation levels following climate stressors [67,68,69].

Exposomes represent a rapidly advancing area that intersects with epigenomics, focusing on thoroughly characterising an individual’s environmental exposures throughout their life [70]. The integration of exposome research with epigenetic studies enhances our comprehensive understanding of the cumulative effects of climate-related factors on the human genome. This approach is facilitated by the development of high-throughput analytical methods and computational frameworks that address the complexity of exposome–epigenome interactions.

Metagenomics has revolutionised microbiota research by enabling researchers to analyse complex microbial communities without cultivating individual species. This is particularly relevant for examining the impact of air pollution on microbiota and human health. High-throughput sequencing techniques, including whole-genome shotgun sequencing and 16S rRNA sequencing, enable scientists to evaluate microbial diversity, abundance, and functional capabilities across different environments, such as the respiratory tract, skin, and gut [71]. Cutting-edge techniques, including metatranscriptomics and metabolomics, enhance metagenomic studies by providing immediate insights into microbial gene expression and metabolic activities under pollution stress. Metatranscriptomics helps identify active microbial genes involved in detoxification and inflammatory responses, while metabolomics analyses microbial metabolites that could serve as biomarkers for health impacts from pollution. Combining multi-omics approaches with metagenomics enhances our understanding of microbiota–host interactions and their impact on pollution-related diseases [72]. All these methods shown in Table 2 highlight the importance of a transdisciplinary approach in investigating the effects of climate change on human biology. Researchers can better understand the mechanisms driving climate-induced molecular changes by merging high-resolution molecular assays with advanced computational analyses and robust epidemiological study designs. These findings have significant implications for public health, as they may identify biomarkers linked to climate stressors, enabling targeted therapeutic and preventive strategies that bolster human resilience to environmental changes.

## 5. Conclusions

In conclusion, we aimed to thoroughly investigate the impact of climate change on human health, with a specific focus on cardiovascular and respiratory diseases. Every 1 °C increase in temperature is associated with a 4.15% increase in cardiovascular mortality and a 2.59% increase in cerebrovascular mortality, particularly in elderly people over 65 years of age [73,74]. We emphasised the potential roles of epigenetic modifications and host–microbiota interactions in the development and progression of CVDs and respiratory diseases. The evidence highlights the complex interplay between environmental factors, epigenetic mechanisms, and microbial populations within the human body. While the studies reviewed offer significant insights, it is essential to acknowledge the contentious nature of some results, which may stem from the complex nature of the research and the numerous factors at play. Additional research is necessary to confirm and build upon these discoveries. Future investigations should emphasise robust methodologies, such as metagenomic and epigenetic approaches, and combine them with other methods to thoroughly clarify the mechanisms by which climate change affects human health. Ultimately, understanding epigenetic and microbiota signatures enables the earlier identification of risk and intervention among vulnerable populations exposed to pollution or extreme temperatures. These insights are especially relevant for guiding surveillance, targeted screening, and adaptation programs in public health settings.

The review highlights how accelerating climate change acts through systemic pathways involving environmental exposures, epigenetic reprogramming, and alterations in the microbiota, thereby increasing the incidence of cardiovascular, respiratory, and NCDs. Our proposal adopts a transdisciplinary framework, distinguishing it from multidisciplinarity (additive juxtaposition of disciplines) and interdisciplinarity (integrated synthesis), to develop surveillance systems that combine real-time environmental data with clinical, molecular, and social determinants to support equitable health adaptation. Research urgently needs to prioritise longitudinal and multicentre studies, embracing “circle management” projects that include populations exposed to varying levels of vulnerability and promote cooperation among disciplines and policymakers. An integrated One Health and targeted public health framework is essential for developing effective prevention and adaptation strategies that can protect the health of the planet. From this perspective, particularly in urbanised contexts, an urgent, collective urban planning strategy is needed to expand green spaces, accelerate the EU’s “Net Zero” target by 2050, improve air quality, and enable the elimination of anthropogenic pressure (Table 3). The pathoclima framework reveals how climate-driven diseases intersect with social vulnerabilities, amplifying health inequalities and necessitating integrated surveillance and policy responses. Epigenetic and microbiota biomarkers offer concrete tools for early detection, risk stratification, and monitoring adaptation effectiveness. These indicators enhance “possible futures” by ensuring sufficient financial resources for climate-health research, targeted legislation, upheld international accords, and ongoing epidemiological data-monitoring systems, and by fostering an educational culture around sustainability and One Health principles.

## Figures and Tables

**Table 1:** Study Identification and Categorization.

Aspect	Details
Databases and Period	PubMed, Scopus; 2019–2025 (focus on recent climate impacts)
Diseases of Interest	Non-communicable diseases (NCDs): cardiovascular and respiratory diseases.
Geographical Scope	Global, with emphasis on high-income regions (Europe, North America, China) for data availability; no strict exclusion, but prioritized peer-reviewed international studies
Inclusion Criteria	English peer-reviewed articles/reviews/e-books on climate factors (air pollution, heatwaves), epigenetics, microbiota, NCD links
Exclusion Criteria	Non-English; pre-2019; unrelated to climate/epigenetics/microbiota; non-peer-reviewed; animal-only studies without human relevance (est. *n* = ~200 excluded via screening)
Search Results	Initial records: ~350 (PubMed ~ 200, Scopus ~ 150); After duplicates/title-abstract screen: ~120; Full-text assessed: ~80; included: 45 studies (reviews *n* = 25, orig. research *n* = 20) + snowballing *n* = 15
Study Categorizations	1. Epigenetics (DNAm clocks, histone changes; *n* = 20); 2. Microbiota (dysbiosis, SCFAs; *n* = 18); 3. Epigenetics and Microbiota changes, NCD pathways *n* = 12; 4. Health outcomes (NCDs, One Health); *n* = 12

**Table 2:** Main methodologies used to study epigenetic alterations and microbiota changes related to climate effects on human health.

Biological Target	Methodology	What It Measures	Typical Application in Climate–Health Studies
DNA Methylation	Whole-Genome Bisulfite Sequencing (WGBS)	Genome-wide DNA methylation at single-base resolution	Detect epigenetic changes associated with heat exposure, air pollution, or environmental stressors
	Reduced Representation Bisulfite Sequencing (RRBS)	DNA methylation in CpG-rich regions	Cost-effective analysis of methylation changes linked to climate-related pollutants
	Illumina Infinium MethylationEPIC BeadChip	Methylation at ~850 k CpG sites	Population studies evaluating environmental and climate exposure effects
	Pyrosequencing	Quantitative methylation of specific CpG sites	Validation of candidate epigenetic markers
Histone Modifications	Chromatin Immunoprecipitation sequencing (ChIP-seq)	Genome-wide histone modification patterns	Study chromatin changes induced by environmental exposures
	ChIP-qPCR	Histone modifications at selected loci	Targeted validation of epigenetic changes
Chromatin Accessibility	ATAC-seq	Open chromatin regions and regulatory elements	Understanding gene regulation changes due to environmental stressors
	DNase-seq	DNA accessibility across the genome	Mapping regulatory regions responsive to environmental stimuli
Non-coding RNA Regulation	RNA sequencing (RNA-seq)	Expression of microRNAs and lncRNAs	Investigating epigenetic regulatory responses to environmental exposures
	qRT-PCR	Quantification of specific miRNAs or lncRNAs	Validation of candidate regulatory RNAs associated with climate stress
Microbiota Composition	16S rRNA gene sequencing	Taxonomic composition of bacterial communities	Assess microbiota changes due to climate-driven factors
	Shotgun metagenomic sequencing	Microbial species and functional genes	Study microbiome functional responses to climate-related exposures
Microbiota Function	Metatranscriptomics	Microbial gene expression	Evaluate functional microbiome changes under environmental stress
	Metabolomics (LC-MS, GC-MS, NMR)	Microbial metabolites	Identify microbiome-derived metabolites affecting host health in response to climate stressors
Host–Microbiome Interaction	Multi-omics integration (epigenomics + metagenomics + metabolomics)	Interactions between microbiota and host epigenetic regulation	Understanding mechanisms linking climate exposure, microbiota, and disease risk
Exposure Assessment	Exposome analysis using wearable sensors, satellite data, and environmental monitoring	Individual-level environmental exposures	Linking climate variables (heat, air pollution, UV radiation) with biological responses

**Table 3:** Proposed Interventions: From Molecular Insights to Implementation.

Level	Intervention	Mechanisms	Implementation Steps	Expected Impact
Individual	Probotic- SCFA supplementation	Counteracts dysbiosis from heat/pollution; restores epigenetic regulation via HDAC inhibition	Prescribe during heatwaves/pollution alerts; RCTs for NCD patients	Low inflammation 15–20%; epigenetic clock slowing
Clinical	Biomarker screening (DNAm clocks + microbiota profiling)	Early detection of accelerated aging/dysbiosis invulnerable population	Integrate into primary care: annual tests for >65yo; NGS/16S kits	Risk stratification; 30% fewer hospitalizations
Community	Urban green spaces + heat early-warning apps	Reduces PM2.5/heat exposure; microbiota stabilization via lower stress	Policy: 30% green cover by 2030; apps linking weather-pollution-health	Low CVD mortality 5–10% during extremes
Policy	One Health surveillance (climate-health dashboards)	Monitors exposome–epigenome-microbiota links; equity-focused	EU-wide: Integrate IPCC/WHO data with epi-surveillance; fund multi-omics cohorts	Adaptation plans; health inequalities low 20%
Research	Longitudinal multi-omics cohorts	Validates biomarkers/interactions for pathoclima	Fund 5-yr studies (*n* > 10k); AI for predictive modeling	Personalized medicine tools by 2030

## Data Availability

All data analysed during this study are included in this article. Further inquiries can be directed to the corresponding author.

## Supplementary Materials

The following supporting information can be downloaded at: https://www.mdpi.com/article/10.3390/ijerph23030388/s1, Table S1: Definition of Common Terms Used in Epigenetics Research; Table S2: Definition of Common Terms Used in Gut Microbiota Research.
