# Supplementary material for: Climate Change, Epigenetics, Microbiota, and Health
Source: Int J Environ Res Public Health. 2026 Mar 18;23(3):388. doi: 10.3390/ijerph23030388 (PMC13027019; doi:10.3390/ijerph23030388)
Supplement: Supplementary file 1 [file ijerph-23-00388-s001.zip › ijerph-4109399-supplementary.pdf]

**Table S1—D**efinition of Common Terms Used in Epigenetics Research.

| Terms | Definition |
|---|---|
| Gene Editing | A technique that allows for the modification of specific DNA sequences in a genome using tools like CRISPR-Cas9. |
| Transcriptome | The complete set of all messenger RNAs (mRNAs) expressed in a cell or tissue at a given time. |
| Proteome | The entire set of proteins expressed by a cell, tissue, or organism at a specific moment. |
| Point Mutation | A change in a single nucleotide base in the DNA that can lead to changes in the protein sequence. |
| Single Nucleotide Polymorphism (SNP) | Genetic variation in a single DNA base that can influence phenotypic traits or disease susceptibility. |
| RNA Interference (RNAi) | A biological process in which small RNAs silence gene expression by interfering with target mRNA. |
| Bioinformatics | A discipline that integrates biology, computer science, and mathematics to analyze complex biological data. |

**Table S2—Definition of Common Terms Used in Gut Microbiota Research.**

| Terms | Definition |
|---|---|
| Microbiota | The community of microorganisms, including bacteria, viruses, fungi, and archaea, that inhabit a specific environment, such as the human gut. |
| Microbiome | The collective genetic material of all the microorganisms in a particular environment, often referring to the gut microbiota. |
| Dysbiosis | An imbalance in the microbial community that can lead to health issues, often associated with diseases like inflammatory bowel disease (IBD). |
| Prebiotics | Non-digestible food components that promote the growth and activity of beneficial gut bacteria. |
| Probiotics | Live microorganisms that, when consumed in adequate amounts, confer health benefits on the host. |
| Short-Chain Fatty Acids (SCFAs) | Fatty acids produced by the fermentation of dietary fibers by gut bacteria, which play a key role in gut and overall health. |
| Fecal Microbiota Transplantation (FMT) | The transfer of stool from a healthy donor to a patient to restore a balanced gut microbiota, often used for treating conditions like recurrent *Clostridioides difficile* infections. |
| Metagenomics | The study of genetic material recovered directly from environmental samples, used to analyze the diversity and function of microbial communities. |
